# Prevalence of overweight preschool children in public day care centers: a cross-sectional study

**DOI:** 10.1590/S1516-31802012000400004

**Published:** 2012-09-04

**Authors:** Viviane Gabriela Nascimento, Janaína Paula Costa da Silva, Ciro João Bertoli, Luiz Carlos Abreu, Vitor Engrácia Valenti, Claudio Leone

**Affiliations:** I PhD. Postdoctoral Researcher in the Department of Maternal and Child Health, Faculdade de Saúde Pública (FSP), Universidade de São Paulo (USP), São Paulo, Brazil.; II MSc. Nutritionist and Doctoral Student of Public Health, Faculdade de Saúde Pública (FSP), Universidade de São Paulo (USP), São Paulo, Brazil.; III PhD. Professor in the Department of Medicine, Universidade de Taubaté (Unitau), Taubaté, São Paulo, Brazil.; IV MD, PhD. Postdoctoral Fellow in the Department of Maternal and Child Health, Faculdade de Saúde Pública (FSP), Universidade de São Paulo (USP), and Head of the Scientific Writing Laboratory, Department of Morphology and Physiology, Faculdade de Medicina do ABC (FMABC), Santo André, São Paulo, Brazil.; V PhD. Student in the Department of Pathology, Faculdade de Medicina da Universidade de São Paulo (FMUSP), São Paulo, Brazil.; VI MD, PhD. Full Professor and Head of the Department of Maternal and Child Health, Faculdade de Saúde Pública (FSP), Universidade de São Paulo (USP), São Paulo, Brazil.

**Keywords:** Child, preschool, Overweight, Body mass index, Child nutrition sciences, Obesity, Pré-escolar, Sobrepeso, Índice de massa corporal, Ciências da nutrição infantil, Obesidade

## Abstract

**CONTEXT AND OBJECTIVE:**

Brazil is undergoing a period of epidemiological transition associated with demographic and nutritional changes. The prevalence of obesity is also increasing in children and is causing numerous health problems that are becoming public health issues. The aim here was to evaluate the prevalence of overweight among children of two and three years of age.

**DESIGN AND SETTING:**

Cross-sectional study in municipal day care centers in Taubaté, state of São Paulo, Brazil.

**METHODS:**

Weight and height measurements were made on 447 preschool children forming a probabilistic randomized sample. Their body mass index (BMI) was calculated. Their nutritional status was classified using the World Health Organization reference cutoff points (2006). Their mean weight, height and BMI were compared according to their age and sex.

**RESULTS:**

The mean values for the final sample (n = 447) were as follows: mean age: 38.6 months (± 3.5) and Z scores for: weight/height (W/H): 0.50 (± 1.22); height/age: -0.03 (± 1.07); weight/age (W/A): 0.51 (± 1.23); and BMI: 0.51(± 1.23). The prevalence of overweight children (BMI > 1 z) was 28.86%, while the prevalence of underweight children (BMI < -2 z) was 0.89%. There were no differences in mean BMI among the two and three-year age groups (P = 0.66).

**CONCLUSION:**

A high prevalence of overweight was observed in the sample of two and three-year-old children, with practically no malnutrition, thus showing that a significant nutritional transition may already be occurring, even in medium-sized cities of developing countries.

## RESUMO

**CONTEXTO E OBJETIVO:**

O Brasil atravessa um período de transição epidemiológica com mudanças demográfica e nutricional. A prevalência de obesidade está aumentando também entre crianças, causando inúmeros problemas de saúde, que estão se tornando questões de saúde pública. Objetivou-se avaliar a prevalência de excesso de peso em crianças de dois e três anos.

**TIPO DE ESTUDO E LOCAL:**

Estudo transversal em creches municipais de Taubaté, São Paulo, Brasil.

**MÉTODOS:**

Em amostra probabilística e randomizada de 447 pré-escolares, foram mensurados peso e altura, e calculado o índice de massa corporal (IMC). O estado nutricional foi classificado segundo pontos de corte do referencial da Organização Mundial de Saúde (2006). Compararam-se os valores médios de peso, estatura e IMC das crianças segundo a idade e sexo.

**RESULTADOS:**

A amostra final (n = 447) apresentou as seguintes médias. Idade: 38,6 meses (± 3,5), escore Z de peso para estatura (P/E): 0,50 (± 1,22), estatura para idade (E/I): -0,03 (± 1,07), peso para idade (P/I): 0,51 (± 1,23) e IMC: 0,51(± 1,23). A prevalência de excesso de peso (IMC > 1 z) foi de 28,86% e de crianças abaixo do peso ideal (IMC < -2 z) foi de 0,89%. Não houve diferença das médias de IMC entre as idades de dois e três anos (P = 0,66).

**CONCLUSÃO:**

Na população de crianças de dois e três anos de idade, já se evidencia elevada prevalência de excesso de peso, praticamente sem desnutrição, indicando que já pode estar ocorrendo uma transição nutricional importante inclusive em cidades médias de países em desenvolvimento.

## INTRODUCTION

Like other developing countries, Brazil is undergoing a period of important epidemiological transition, which has been associated with both demographic and nutritional changes. This transition has resulted in reductions in the prevalence of malnutrition, with a subsequent increase in the prevalence of obesity, rising to epidemic proportions.^1^ Obesity in children is increasing rapidly and is causing numerous health problems that are becoming public health issues, both in developed and in developing countries.^2^

Overall, the prevalence of obesity in Brazil increased by approximately 53% from the 1970s to the latter part of the 20^th^ century.^3^ It is of great concern that the rise in the numbers of overweight and obese individuals has been observed starting at very early ages and across all socioeconomic levels,^4^^,^^5^ including preschool children.^6^ Early evaluation of the presence of overweight children is important, since this allows preventive measures to be taken.^7^ More specifically, detection of changes in body composition during early childhood allows early interventions to be set in motion, which may aid in preventing the development of long-term comorbidities resulting from obesity.^8^

## OBJECTIVE

To evaluate the prevalence of overweight and obese children of two and three years of age who were attending day care centers in a medium-sized city in the state of São Paulo.

## METHODS

This was a cross-sectional study on a randomized sample of two to three-year-old children who were enrolled in Maternal I classes in day care centers in the city of Taubaté, state of São Paulo, Brazil, in 2009.

The sampling used conglomerates; the sampling unit was the day care center, and a probabilistic and random draw was carried out based on the total of 59 public daycare centers belonging to the Department of Education and Culture of the city of Taubaté. The day care centers were randomly assigned in a sequential manner to complete the sample of children needed, which was calculated as being 408 children (considering 5% alpha, a test power of 90%, and a prevalence of overweight of at least 25%). This yielded an acceptable standard error of 10% (2.5 percentage points) in the forecasted prevalence. The first nine randomly assigned day care centers allowed us to reach the final sample size of 447 children who were enrolled in the study. The sample size was calculated using the PS Power and Sample Size Calculations 2.1.30 software.

The exclusion criteria for the study were as follows: children with chronic or specific growth diseases; children who did not attend preschool on the days scheduled; and children whose parents did not allow them to participate. We included only children who were enrolled and were attending Maternal I classes. Anthropometric data were collected in April 2009.

Portable electronic scales (Seca - 150 kg and 10-g accuracy) were used for weighing the children. The children were weighed wearing light clothes and without their shoes. For height measurements, we used a portable, wall-mounted stadiometer (Wiso). The children stood with their heels, calves, buttocks, and shoulders touching the wall and placed their head against the wall in accordance with the Frankfurt plan. All anthropometric measurements were obtained using the methods described by Lohman et al.^9^

We calculated the body mass index (BMI) from the weight and height measurements. To sort the overweight individuals, the weight, height and BMI values were transformed into Z scores in accordance with the World Health Organization references (WHO) of 2006.^10^ Next, we compared the children’s mean values for weight, height and BMI according to their age and sex. The classification proposed by the Brazilian Ministry of Health^11^ was used to classify nutritional status and height. We took the category of overweight children to include not only those who were already classified as overweight and obese, but also children who were defined according to the Ministry’s classification as being at risk of overweight (BMI > 1 Z score).

For data analysis, we used the Student t test to compare the means of independent samples, with a significance level of a = 5%, using the Statistical Package for the Social Sciences (SPSS) 12.0 software.

The present study was approved by the Research Ethics Committee of the School of Public Health, Universidade de São Paulo (Protocol 1877, April 2009). A free and informed consent form was sent to the mothers or guardians by the day care centers and was filled in and signed before data collection proceeded.

## RESULTS

The final sample consisted of 447 children, of whom 220 (49.2%) were female and 227 (50.8%) were male. Of the 447 children, 115 (25.7%) were younger than 36 months (mean age 34.1 ± 1.44 months), and 331 (74.3%) were older (mean age 40.2 ± 2.52).

The children aged less than 36 months presented the following mean anthropometric Z scores: weight for height (W/H): 0.50 (± 1.03); height for age (H/A): 0.00 (± 1.09); weight for age (W/A): 0.51 (± 1.23); and BMI 0.60 (± 1.03). The children aged over 36 months presented a Z score of W/H mean values of: 0.50 (± 1.28); H/A: -0.10 (± 1.06); W/A: 0.30 (± 1.23); BMI: 0.50 (± 1.29). There were no significant differences in the means of the various anthropometric parameters among the two and three-year age groups (P > 0.05).

Table 1 shows that the prevalence of overweight (> 1 Z score of BMI) was 28.86%, while less than 1% of the children had some degree of underweight.

**Table 1.** Frequency distribution of the preschool children in the city of Taubaté, state of São Paulo, Brazil, 2009, according to their nutritional status, as defined by their body mass index

Nutritional status	n	%
Marked underweight	1	0.22
Underweight	3	0.67
Well-nourished	314	70.25
Risk of overweight	89	19.91
Overweight	22	4.92
Obesity	18	4.03
Total	447	100.00

Table 2 shows that less than 3% of the children had short stature.

**Table 2.** Frequency distribution of the preschool children in the city of Taubaté, state of São Paulo, Brazil, 2009, according to their height for age

Height for age	n	%
Very low height for age	-	-
Low height	13	2.91
Appropriate height	434	97.09
Total	447	100.00

Based on the Z scores in Table 3, the average for the various parameters was higher than for the reference value used, with the exception of height for age, which was almost identical to the reference index.

**Table 3.** Z score distribution of weight, height and body mass index (BMI) among preschool children in the city of Taubaté, state of São Paulo, Brazil, 2009

Z scores	Mean	Standard deviation	Median
Weight/height	0.50	? 1.22	0.46
Height/age	- 0.03	? 1.07	0.09
Weight/age	0.33	? 1.19	0.25
BMI/age	0.51	? 1.23	0.42

The analysis according to sex showed a growth pattern that was practically identical for the two sexes (Table 4).

**Table 4.** Z score distribution of weight, height and body mass index (BMI) among preschool children in the city of Taubaté, state of São Paulo, Brazil, 2009, according to their sex

Z scores	Female			Male		
	Mean	Standard deviation	Median	Mean	Standard deviation	Median
Weight/height	0.5	? 1.2	0.5	0.5	? 1.3	0.4
Height/age	- 0.1	? 1.0	0.0	0.0	? 1.1	- 0.1
Weight/age	0.3	? 1.2	0.3	0.3	? 1.2	0.2
BMI/age	0.5	? 1.2	0.5	0.5	? 1.3	0.4

## DISCUSSION

The average physical growth rate of children within a population is known to be a useful indicator of the health and wellbeing of that population.^12^ The average height found among the preschool children that we sampled in this study showed that their growth was adequate and was consistent with the World Health Organization reference.^10^ However, the same was not found in relation to weight, which, according to the three parameters analyzed, was on average above the reference index. Of greater importance was the early onset of overweight among these children, which in our study was shown by the fact that a population of children of less than four years of age was already presenting nearly twice the expected prevalence, based on the reference index. There were no significant differences when the parameters were analyzed according to gender. The observed prevalence in our study (28.9% presenting overweight) was higher than what was reported by Rodrigues et al.^13^ in 1989, among Brazilian children less than four years of age. Recently, in Mexico, Fernald and Neufeld also observed a high prevalence of overweight among preschool children aged two and three years: respectively 19.8% and 23.7%.^14^

The adequate structural growth associated with high prevalence of overweight observed in this study characterizes the presence of a state of nutritional and epidemiological transition, thus indicating that Taubaté children have access to a satisfactory amount of food but that the diet may be of low quality. Since healthy food is less available to individuals of lower socioeconomic classes, an increase in the numbers of overweight individuals may occur. The relationship between obesity and low socioeconomic status has also been observed in other developing countries.^15^

According to data from the National Health and Nutrition Survey (Pesquisa Nacional sobre Saúde e Nutrição, PNSN, 1989)^16^ obtained about 20 years ago, the prevalence of overweight in Brazil was already approximately 2.5% among children in families of lower income and 8% among children of higher income. Thus, there was already evidence of overweight among children in the first years of life, in the Brazilian population.

Several studies have explored the prevalence of overweight young children according to socioeconomic status. Santos and Leão^17^ described the anthropometric profile of preschool children enrolled in day care centers in Rio de Janeiro, and observed that in low-income populations, the prevalence of overweight children was 27.3%. In a study aiming to determine the prevalence of overweight among 3,627 preschool children in the city of Natal, it was found that the prevalence was 19.7% in public schools and 32.5% in private ones.^18^ In a study that sought to examine socioeconomic factors and other conditions of family life that were associated with being overweight, Vitolo et al.^19^ found a prevalence of 9.8% among 3,957 children between the ages of 1 and 60 months. In the same study, the prevalence of overweight children in the higher socioeconomic class was 13.1% and in the remaining classes, it was 9.3%.

In general, higher prevalence of obesity was observed among children of higher socioeconomic status. In a study conducted among 230 private preschool children in the city of Recife, the prevalence of overweight children was 33.9%.^20^ Similarly, in a study by Simon et al.,^21^ the prevalence of overweight among private preschool children in the city of São Paulo was 34.4%.

Comparisons with data from the literature should be made carefully, since there are many differences in the methods used, particularly with regard to the reference index, age and the socioeconomic level of the study populations. Most of the studies mentioned in this paper described the prevalence of overweight among children between the ages of two and six years, thus including children older than those evaluated here. This is relevant, since older children would have had more time to increase their weight than would the sample we studied. The prevalence of overweight among the children in our study was very similar to values observed in other studies in the literature that evaluated children under four years of age. Since our sample consisted of children who were just beginning preschool, we assumed that they would show a lower prevalence of overweight, which was not the case. This is particularly of concern, because it demonstrates that the rate of presenting a risk of overweight was also higher at earlier ages.

According to the reference used for classifying overweight children, variations that may result in higher or lower prevalence can occur. According to Torres et al.,^22^ from using the new World Health Organization reference index, the prevalence of overweight is higher than when using the NCHS/CDC reference index of 2000. Another study that aimed to compare the classification criteria for the nutritional status of preschool children at public day care centers in São Paulo also showed that the prevalence of overweight children is higher when adopting the WHO reference index.^23^ This could also explain, at least in part, the differences in prevalence found between the literature and this study.

Comparing different socioeconomic levels, the differences seem to be decreasing, given that similar prevalences of overweight are observed regardless of socioeconomic status. This suggests that nowadays, higher prevalence of overweight might not be directly linked to socioeconomic conditions.

The existence of the overweight phenomenon at early preschool ages, as observed in this study, even among low-income populations that have virtually no malnutrition, indicates that an important nutritional transition is occurring, even in medium-sized cities of developing countries, i.e. not only in large metropolitan areas. Another study on preschool children in a midsize Brazilian city also pointed towards the existence of a nutritional transition in Brazil.^24^

This scenario highlights another point: the excessive increase in weight is beginning in children as young as two years of age. This may be due to the type and quantity of food that the child receives during the two first years of life. This hypothesis is even more plausible when taking into consideration studies that show that the duration of breastfeeding in Brazil is shorter than what is recommended by the World Health Organization.^25^^,^^26^^,^^27^ Recent studies have shown that the duration of exclusive breastfeeding is short, with averages of 1.5 months^28^ and less than 3 months.^29^

According to Koletzko et al.,^30^ adequate duration of breastfeeding after birth is associated with low prevalence of overweight or obesity at school age. This protective effect of breastfeeding is independent of social status and lifestyle differences. In another study,^31^ the same authors showed that protein consumption in early childhood may influence weight gain, thus indicating that the high amount of protein contained in infant formulas would be associated with an excessive increase in weight during the first two years of life and that the same effect does not occur for height growth.^30^^,^^31^ Rapid weight gain in early childhood is a risk factor for adult adiposity and obesity. Early intervention could prevent the risk of obesity in adulthood.^32^

## CONCLUSION

In view of these assumptions and the results observed from this study, there is an urgent need for early interventions to avoid occurrences of excessive weight gain in early life. This may be a more effective way of preventing the emergence of overweight and its associated comorbidities, which are already present among young adults.
